# Supplementary material for: Morphogenesis of liquid crystal topological defects during the nematic-smectic A phase transition
Source: Nat Commun. 2017 May 30;8:15453. doi: 10.1038/ncomms15453 (PMC5459947; doi:10.1038/ncomms15453)
Supplement: Supplementary Information — Supplementary Figures [file ncomms15453-s2.pdf]

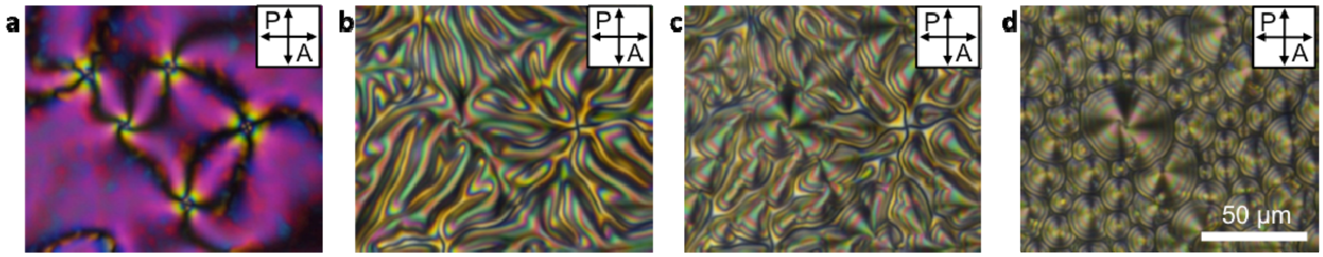

Supplementary Figure 1. POM images of defects during the N-SmA phase transition in a sandwiched cell with hybrid anchoring. **a**, N phase. **b**, Vicinity of the SmA phase, **c-d**, SmA phase. The cell exhibits hybrid anchoring and has a thickness of 5 μm (top interface: vertical alignment, bottom substrate: planar alignment).

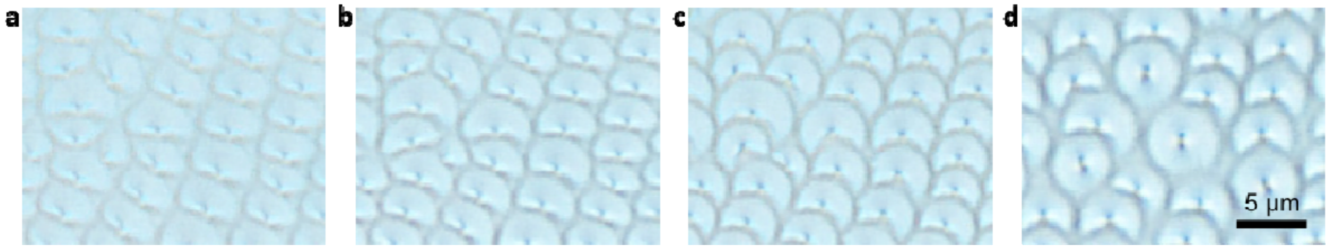

Supplementary Figure 2. Optical microscopic images of the FCD-TFCD transition, as the temperature is decreased from 32.1 ^{o}C to 31.8 ^{o}C, illustrating the incompleteness of the domains.
